# A Cross-Sectional Survey of Knowledge, Attitude and Practices Related to Cutaneous Leishmaniasis and Sand Flies in Punjab, Pakistan

**DOI:** 10.1371/journal.pone.0130929

**Published:** 2015-06-19

**Authors:** Ayesha Akram, Hafiz Azhar Ali Khan, Abdul Qadir, Arshad Makhdoom Sabir

**Affiliations:** 1 College of Earth and Environmental Science, University of the Punjab, Lahore, Pakistan; 2 Institute of Agricultural Sciences, University of the Punjab, Lahore, Pakistan; 3 Rice Research Institute, Kala Shah Kaku, Pakistan; Institut Pasteur, FRANCE

## Abstract

**Background:**

Recent outbreaks of cutaneous leishmaniasis make the disease a public health concern in Punjab, Pakistan. The knowledge of how the population perceives the disease and its vector is essential in order to design an effective management strategy, but such studies are rare in Pakistan.

**Methodology/Principal Findings:**

The present study was based on a cross-sectional self-administered survey comprising 250 household samples collected from five localities including Bhawalpur, Multan, Jhang, Faisalabad and Lahore. The results revealed that the respondents had a poor knowledge of the vector and disease. Few of the respondents were aware about the identification of sand flies, their breeding place, biting time, transmission of leishmaniasis and control measures. Skin infection and sandflies as the main disease symptom and vector of the disease, respectively, were known to some of the respondents. Some believed that summer was the main peak incidence of the disease and it could be transmitted from man to man via contact. However, most of the respondents believed that the disease could be cured. Admission to hospitals, cleanliness and use of bed nets were the treatment measures for the disease in suspected patients, whereas some thought that the use of bed nets could be helpful in preventing leishmaniasis infection.

**Conclusions/Significance:**

Poor knowledge of the disease and its vector in the study population emphasize the need to initiate health education and awareness campaigns to minimize the risks of cutaneous leishmaniasis outbreaks in the future.

## Introduction

Cutaneous leishmaniasis is a sand fly-borne neglected tropical disease caused by protozoan parasites in the genus *Leishmania*. It is commonly known as *post-kala-azar dermal leishmainiasis* in Hindi and *lahori phora* or *sehrai phora* in Urdu. The disease is commonly characterized with skin sores or skin infection symptoms. About one million cases of cutaneous leishmaniasis occur annually worldwide with the hotspots in Afghanistan, Algeria, Iran, Pakistan, Peru, Brazil, Saudi Arabia, Colombia and Tunisia [1]. In Pakistan, it is one of the major and rapidly increasing public health issues, especially alongside regions bordering the neighboring Afghanistan and cities that have had the maximum influx of refugees from Afghanistan. About 5000 cases of cutaneous leishmaniasis had been reported during 2002, in the Khyber Pakhtun Khwa province of Pakistan [2]. This disease is highly endemic in different parts of the country, including the Punjab province, but recently it seems to become an epidemic [3] and hence disease alerts have been generated in the country through print media [4].

The diversity of leishmania strains involved in cutaneous leishmaniasis and unavailability of universally acceptable, safe, and effective vaccine make the treatment of patients difficult [5], and the disease continues to engulf new regions. In this scenario, personal protection and source reduction of the vector remained an effective tool in the prevention of cutaneous leishmaniasis [6]. However, the adoption of preventive measures strongly depends on the attitudes and behaviors of the population at risk. Therefore, in order to control leishmaniasis, it is essential to know the risk factors associated with it, and to understand the disease-related knowledge, attitudes, and practices (KAP) of the population [7]. Studies in Colombia revealed a direct relationship between awareness of the population at risk and adoption of preventive measures [8]. Such studies helped the health education policy makers to implement effective disease control programs [9]. Therefore, the present cross-sectional survey study was designed with the focus on to assess the level of knowledge, attitude and practices of the community related to cutaneous leishmaniasis. To date, such KAP studies are rare at Pakistan level, and thus this study presents the information on KAP related to cutaneous leishmaniasis.

## Methods

### Ethics statement

The study and the verbal consent process were approved by the doctoral research committee of the College of Earth and Environmental Science, University of the Punjab, Lahore. Verbal informed consent was obtained before the start of interviews and only those individuals were selected who showed their willingness to participate in the survey. Verbal consent was used to ensure anonymity and accommodate illiterate study subjects.

### Study sites and survey

The study was carried out in five localities of the Punjab province, Pakistan including Bahawalpur (29.3956° N, 71.6836° E), Multan (30.1978° N, 71.4697° E), Jhang (30.5833° N, 71.6500° E), Faisalabad (31.4180° N, 73.0790° E) and Lahore (31.5497° N, 74.3436° E). Punjab is the most populated province of Pakistan, which accommodates almost 50% of the country’s population. Study areas had three major seasons: a hot season usually during the months of April-June, when the mercury rises as high as 110°F, a rainy season usually during the months of July-September, with an average annual rainfall of 46 cm in the plains, and a mild season during the rest of the year with the mercury goes down as low as 40°F [9, 10].

The survey was carried out by following the methodology of Khan et al [9]. Briefly, a questionnaire was developed by following Frary’s guidelines on questionnaire construction [11]. The questionnaire was consisted of three main parts: socio-demographic characteristics of the participants, knowledge of sand flies and leishmaniasis, and attitudes and practices related to leishmaniasis. Most of the questions were open ended and the participants were selected based on convenience sampling, and their willingness to participate. Knowledge of sand flies was assessed by asking questions related to the disease spread, breeding habitats, biting time and control measures. Knowledge of leishmaniasis was assessed by asking questions like symptoms and vector of the disease, peak incidence time, etc. Whereas, attitudes and practices were assessed by the responses of the respondents related to the seriousness of the disease, patient care, and preventive measures of the disease and information source. All the collected data were analyzed by descriptive statistics using the software SPSS v16.0.

## Results and Discussion

The present survey reports on knowledge, attitude and practices of individuals from five localities in the Punjab province, Pakistan. Though there can be no certainty that all respondents in such type of surveys will do what they say, it gives insights into how they are thinking about what they do [12].

### Socio-demographic characteristics of the respondents

In the present study, 250 individuals were interviewed from five localities of the province Punjab. Of these, 53.6% were females while 46.4% were male (Table 1). Most of the respondents (65.2%) were in the age group 16–28 years, while 21.2% were in the 29–40 years age group. The education characteristics of the studied population revealed that most of the respondents were graduates while 10% had no education. Regarding occupation of the studied population, farmers, teachers and doctors constituted 10.8, 8.4 and 6% respectively, whereas 74.8% were in the category of others including shopkeepers, businessmen, students, housewives, elderly persons unable to work for a living. Of the total household, 50% had six to nine members at their homes, 35.2% had less than five members while 14.45 had ≥10 members.

**Table 1:** Socio-demographic characteristics of interviewee in five localities of the province Punjab (n = 250).

Characteristics	Categories	Multan n (%)	Bahawalpur n (%)	Faisalabad n (%)	Jhang n (%)	Lahore n (%)	Total n (%)
Gender							
	Male	25(50)	21(42)	24(48)	24(48)	22(44)	116(46.4)
	Female	25(50)	29(58)	26(52)	26(52)	28(56)	134(53.6)
Age							
	16–28	34(68)	25(50)	36(72)	33(66)	35(70)	163(65.2)
	29–40	6(12)	16(32)	10(20)	13(26)	8(16)	53(21.2)
	> 40	10(20)	9(18)	4(8)	4(8)	7(14)	34(13.6)
Education							
	Illiterate	3(6)	10(20)	7(14)	1(2)	4(8)	25(10)
	Primary	5(10)	8((16)	3(6)	2(4)	2(4)	20(8)
	Secondary	8(16)	11(22)	7(14)	6(12)	6(12)	38(15.2)
	>Graduation	34(68)	21(42)	33(66)	41(82)	38(76)	167(66.8)
Occupation							
	Farmer	10(20)	6(12)	1(2)	-	4(8)	21(8.4)
	Teacher	7(14)	3(6)	4(8)	6(12)	7(14)	27(10.8)
	Doctor	2(4)	9(18)	1(2)	2(4)	1(2)	15(6)
	Others	31(62)	32(64)	44(88)	42(84)	38(76)	187(74.8)
Household members							
	≤5	24(48)	10(20)	16(32)	20(40)	18(36)	88(35.2)
	6–9	21(42)	28(56)	25(50)	23(46)	28(56)	125(50)
	≥10	5(10)	12(24)	9(18)	6(12)	4(8)	36(14.4)

### Knowledge about sand flies and leishmaniasis

Knowledge of the respondents regarding sand flies is shown in the Table 2. The study revealed that 20% of the respondents were able to differentiate sand flies from common house flies and mosquitoes, whereas 80% had no idea of identification. When the respondents were asked “do sand flies transmit diseases? If yes, name any of the disease”, most of the respondents (84%) answered “I don’t know”, while 9.2% answered that sand flies transmit leishmaniasis. 1.2% of the respondents thought that sand flies transmit diseases, but they failed to name any of the disease. For the breeding places of sand flies, 18.4% thought that unhygienic conditions are suitable breeding places, whereas 10.8%, 6.4% and 4.8% said moist places, fresh water and hospitals waste disposal sites, respectively. However, 59.6% had lack of information regarding breeding places of sand flies. Regarding the biting time of sand flies, 14% thought that it bites during dusk and dawn, followed by at any time of the day (13.2%), during daytime (10%), during midnight (8%), whereas the rest of the respondents (54.8%) were unaware. For the control measures of sand flies, 13.2% were in the opinion that sand flies can be controlled by meshing of doors and windows. The use of insect repellents, sanitation, insecticide sprays and fly papers were also answered by some of the respondents, whereas 50% were unaware about the control measures. The results revealed poor knowledge of the respondents related to sand flies and their control measures. In contrast, the survey conducted by Singh et al. [13] in India revealed that most of the people were aware of the biting time of sand flies and subsequent control and preventive measures. It is important for communities to know the breeding habitats, biting mode or time and control measures to minimize the chances of vector- human contact [13]. However, the majority of the respondents were unaware of these facts. One probable reason for this could be that these flies are nocturnal in habits [14] which make it difficult for the people to recognize their habits.

**Table 2:** Knowledge of the respondents related to sand flies (n = 250).

Characteristics	Categories	Number	%
Can you identify/differentiate sandflies from common house flies and mosquitoes?			
	Yes	50	20
	No	200	80
Do sand flies transmit diseases? If yes, name any of the disease			
	Allergy	3	1.2
	Cough	1	0.4
	Diarrhea	2	0.8
	Digestive problems	1	0.4
	Fever	3	1.2
	Leishmaniasis	23	9.2
	Similar to dengue	1	0.4
	Skin diseases	1	0.4
	sneezing	1	0.4
	Yes, but don’t know any name of the disease	3	1.2
	I don’t know	210	84
Do you know breeding places of sand flies?			
	Hospitals waste disposal sites	12	4.8
	Unhygienic conditions	46	18.4
	Fresh water	16	6.4
	Moist places	27	10.8
	I don’t know	149	59.6
Do you know biting time of sand flies?			
	During dusk and dawn	35	14
	During midnight	20	8
	During day time	25	10
	At any time	33	13.2
	I don’t know	137	54.8
Do you know the control methods of sand flies?			
	Fly paper	14	5.6
	Insecticide sprays	20	8
	Insect repellents	29	11.6
	Sanitation	21	8.4
	Meshing of the doors and windows	33	13.2
	Other	8	3.2
	I don’t know	125	50

Knowledge of the respondents regarding leishmaniasis is shown in the Table 3. About 24.4% of the respondent saw its case, whereas 75.6% had no such experience. When the respondents were asked about the signs/symptoms of the disease, 33.6% said skin infection is the main symptom of leishmaniasis. Some of the respondents also answered fever, enlargement of liver and spleen and anemia as the symptoms, whereas the majority of the respondents (56%) were unaware. Most of the respondents (57.6%) were unaware of the vector of leishmaniasis; however, some of the respondents answered sand flies (27.6%), insects (7.6%) and house flies (2%) as vectors of the disease. 34.4% of the respondents thought that it can be transmitted from man to man via contact, whereas 65.6% were against this opinion. According to the respondents, summer (24.8%), spring (8.4%) and winter (6%) were the peak occurrence time of the disease; whereas 60.8% answered “I don’t know”. However, the majority of the respondents (70.4%) thought that the disease is curable while the rest of the respondents answered “no”.

**Table 3:** Knowledge of the respondents related to cutaneous leishmaniasis (n = 250).

Characteristics	Categories	Number	%
Did you ever see a leishmaniasis patient or have a history of leishmaniasis?			
	Yes	61	24.4
	No	189	75.6
Do you know symptoms of the disease?			
	Skin infection	84	33.6
	Fever	11	4.4
	Enlargement of liver and spleen	12	4.8
	Anemia	3	1.2
	I don’t know	140	56
Do you know the vector of the disease?			
	Sand fly	69	27.6
	House fly	5	2
	Insects	19	7.6
	others	13	5.2
	I don’t know	144	57.6
Can it be transmitted from man to man?			
	Yes	86	34.4
	No	164	65.6
Peak incidence time?			
	Summer	62	24.8
	Winter	15	6
	Spring	21	8.4
	I don’t know	152	60.8
Is leishmaniasis curable?			
	Yes	176	70.4
	No	74	29.6

Understanding local knowledge of the communities with respect to health issues can be an important tool, particularly in areas like Pakistan where the quality of biomedical healthcare is lacking [9]. Therefore, assessment of local knowledge could be helpful in fulfilling the gap between the need of health facilities and their provision *in situ* [15]. Despite the fact of being sand flies very serious insect vectors of leishmaniasis, few people were aware of their relationship in causing infection. Lack of disease and its vector’s knowledge is a matter of concern for the adoption of preventive measures, and to seek early treatment [9]. In contrast, the people from epidemic areas of India [13] and Bangladesh [16] had good knowledge about leishmaniasis.

### Attitudes and practices related to cutaneous leishmaniasis

Majority of the respondents (58%) thought that leishmaniasis was less serious than dengue fever while 42% respondents were against this opinion (Table 4). The most probable reason might be due to the fact that Punjab province had faced more serious epidemics of dengue fever in recent years [17] as compared to leishmaniasis epidemics. In this scenario, it is expected that the people would consider it a less severe disease. When the respondents were asked “how patient care with leishmaniasis should be done?”, patient admission to the hospital was the answer of 31.2% respondents followed by cleanliness (27.2%), use of bed nets (26%), isolation of patients (9.2%) and precaution in diet (6.4%). The use of bed nets to control leishmaniasis was the opinion of 9.6% of the subjects, whereas the majority of the respondents (80.8%) were unaware about the preventive measures for leishmaniasis. Regarding the source of information related to sand flies and leishmaniasis relationship, friend, neighbor or teacher was the answer of 40.8% of the subjects followed by television (34.8%), print media (15.2%) and radio (9.2%) (Table 4). Health education interventions in teaching and electronic media can be a successful leishmaniasis information tool [18]. Recently, information related to dengue fever and it vector mosquitoes have been added in the school curricula after severe epidemics of dengue fever in Punjab, Pakistan [9]. Similarly, knowledge of leishmaniasis and its vector may also be included in the curricula as an educational campaign. The addition of such type of knowledge in curricula or health education magazines, particularly in the form of poems, stories, and/or folk songs could be helpful for the well-being of new generations and can put scientific learning within its traditional context [19, 20].

**Table 4:** Attitude and practices of the respondents related to cutaneous leishmaniasis (n = 250).

Characteristics	Categories	Number	%
Seriousness of the disease as compared to dengue fever			
	Leishmaniasis is more serious than dengue	105	42
	Dengue is more serious than leishmaniasis	145	58
Patient care			
	Cleanliness	68	27.2
	Use of bed net	65	26
	Isolation of patient	23	9.2
	Precaution in diet	16	6.4
	Admission to Hospital	78	31.2
Preventive measures for leishmaniasis			
	Avoid dirt	2	0.8
	Avoid unhygienic conditions	2	0.8
	Bed net	24	9.6
	Cleanliness	9	3.6
	Sanitation	3	1.2
	Health education	2	0.8
	Insect repellents	6	2.4
	Don’t know	202	80.8
Information source			
	Radio	23	9.2
	Television	87	34.8
	Print media	38	15.2
	Friend/neighbor/teacher	102	40.8

### Limitations

The use of convenience sampling makes the generalizability of the study limited. However, considering the study’s constraints of time and cost, the above said sampling scheme was more feasible than probability sampling. Despite limitations, the findings of the present survey have important implications for the management of sand flies and leishmaniasis, and to emphasize the need to initiate health education campaigns.

## Conclusion

The findings of the present survey revealed lack of knowledge among communities related to sand flies, leishmaniasis, their relationship in causing infection and its management. These findings also emphasize the need for health education campaigns to create awareness among communities about this disease and its vector and management practices to minimize its incidence.
